# Should We Always Perform Preoperative Chest Computed Tomography in Patients with cT1a Renal Cell Carcinoma?

**DOI:** 10.3390/cancers14225558

**Published:** 2022-11-12

**Authors:** Jae-Wook Chung, Jun-Koo Kang, Se Won Jang, Eun Hye Lee, So Young Chun, Seock Hwan Choi, Jun Nyung Lee, Bum Soo Kim, Hyun Tae Kim, See Hyung Kim, Tae-Hwan Kim, Eun Sang Yoo, Tae Gyun Kwon, Dong Jin Park, Yun-Sok Ha

**Affiliations:** 1 Department of Urology, Kyungpook National University Hospital, Daegu 41944, Korea; 2 Biomedical Research Institute, Kyungpook National University, Daegu 41944, Korea; 3 Department of Urology, School of Medicine, Kyungpook National University, Daegu 41944, Korea; 4 Joint Institute for Regenerative Medicine, Kyungpook National University, Daegu 41940, Korea; 5 Department of Radiology, School of Medicine, Kyungpook National University, Daegu 41944, Korea; 6 Department of Urology, Dongguk University College of Medicine, Gyeongju 38066, Korea

**Keywords:** renal cell carcinoma, clinical staging, lung metastasis, chest computed tomography scan

## Abstract

**Simple Summary:**

We aimed to establish an objective standard for optimal timing of preoperative chest computed tomography (CT) in patients with renal cell carcinoma (RCC). The overall rate of positive chest CT scans before nephrectomy was 3.03% (27/890). Only one patient had lung metastasis before surgery for cT1a. cT stage (≥cT1b), Charlson comorbidity index (CCI) ≥4, and low albumin/globulin ratio (AGR) were associated with a higher risk of positive chest CT scans. After 890-sample bootstrap validation, the concordance index was 0.80. The net benefit of the proposed strategy was superior to that of the select-all and select-none strategies according to decision curve analysis. Therefore, when chest CT was performed with a risk of a positive result ≥10%, 532 (59.8%) negative chest CT scans could be prevented. Only 24 (2.7%) potentially positive chest CT scans were misdiagnosed. Therefore, we recommend chest CT in patients with ≥cT1b disease, CCI ≥4, and low AGR.

**Abstract:**

No definitive criteria regarding the performance of preoperative chest computed tomography (CT) in patients with cT1a renal cell carcinoma (RCC) exists. We aimed to establish an objective standard for the optimal timing of preoperative chest CT in patients with RCC. Data from 890 patients who underwent surgical treatment for RCC between January 2011 and December 2020 were retrospectively collected. The primary endpoint was detection of lung metastasis on chest CT before nephrectomy. A multivariable logistic regression model predicting positive chest CT scans was used. Predictors included preoperative cTN stage, presence of systemic symptoms, Charlson comorbidity index (CCI), platelet count/hemoglobin ratio, albumin/globulin ratio (AGR), and De Ritis ratio. The overall rate of positive chest CT scans before nephrectomy was 3.03% (27/890). Only one patient had lung metastasis before surgery for cT1a. cT stage (≥cT1b), CCI ≥4, and low AGR were associated with a higher risk of positive chest CT scans. The best cutoff value for AGR was 1.39. After 890-sample bootstrap validation, the concordance index was 0.80. The net benefit of the proposed strategy was superior to that of the select-all and select-none strategies according to decision curve analysis. Therefore, when chest CT scans were performed with a risk of a positive result ≥10%, 532 (59.8%) negative chest CT scans could be prevented. Only 24 (2.7%) potentially positive chest CT scans were misdiagnosed. Therefore, we recommend chest CT in patients with ≥cT1b disease, CCI ≥4, and low AGR.

## 1. Introduction

Malignant neoplasm of the kidney is the third most common urologic malignancy after prostate and bladder cancers [1]. Renal cell carcinoma (RCC) accounts for 80–85% of all kidney cancers [2]. In 2021, the estimated number of cases of newly diagnosed kidney cancer in South Korea was 6244 [3]. In South Korea, the 5-year survival rate of patients with kidney cancer has been increasing over the last few decades [4].

RCC is occasionally diagnosed with synchronous metastasis in 10–20% of cases [5]. The lungs are the most frequent sites of metastasis [6,7]. The European Association of Urology (EAU) and National Comprehensive Cancer Network (NCCN) guidelines recommend chest computed tomography (CT) for the staging of patients diagnosed with RCC [8,9]. However, the initial and follow-up imaging protocols for lung metastasis are unclear [10]. Moreover, the characteristics of lung metastasis and necessity of chest CT for detecting recurrence after initial curative nephrectomy are uncertain [11]. Therefore, the imaging modalities for initial optimal workup, follow-up interval, and duration vary.

Indiscriminate and excessive scanning during chest CT can increase radiation exposure and medical insurance expenditure [12]. With the widespread application of cross-sectional abdominal CT, most cases of RCC are incidentally found at an early stage and have a low risk of metastasis recurrence after curative nephrectomy [13]. Furthermore, in a few selected patients with favorable clinical characteristics of RCC at initial diagnosis, preoperative chest CT can be omitted because of a low risk of pulmonary metastasis from RCC [12]. Therefore, we hypothesized that not all patients with low-stage RCC would require chest CT before nephrectomy. 

To date, there are no specific criteria or objective standards indicating which patients with RCC should undergo preoperative chest CT. Many urologists decide when to perform chest CT for RCC staging based only on empirical experiences and processes owing to the absence of an appropriate predictive model to assess the risk of lung metastasis in patients with RCC. Therefore, this retrospective, single-center study with a relatively large cohort aimed to predict and select low-risk patients for whom staging chest CT for RCC can be safely omitted. 

## 2. Materials and Methods

### 2.1. Study Design

Data from 1136 patients who had previously undergone radical or partial nephrectomy for renal masses at Kyungpook National University Hospital between January 2011 and December 2020 were analyzed. All patients underwent abdomen/chest CT preoperatively. Brain CT was performed only if symptoms, such as headache, were present.

Patients whose pathological results were benign renal masses were excluded (n = 80). Patients with multiple metastases to the lungs and viscera or bone, which were revealed during preoperative abdomen/chest CT or bone scans, were excluded (n = 92). Patients with bilateral disease (n = 20), von Hippel–Lindau disease (n = 2), and chronic kidney disease (serum creatinine level ≥2.0 mg/dL; n = 30) were also excluded. Further, patients with previously diagnosed hepatic or hematologic diseases (n = 22) were excluded. Blood tests, including those for estimating platelet (PLT) count and serum hemoglobin (Hb), albumin, globulin, aspartate transaminase (AST), and alanine aminotransferase (ALT) levels, were performed within at least 1 week after surgery.

The follow-up regimen included blood and urine tests and CT. Imaging analyses, including chest and abdominal CT, were performed at 3, 6, and 12 months postoperatively; every 6 months from 1 to 5 years; and annually thereafter.

The primary endpoint was detection of lung metastasis on preoperative chest CT, defined as ≥1 lesion in the lung parenchyma suspected to be metastasis. The secondary endpoint was detection of lung metastasis on postoperative chest CT. Lung metastasis was clinically diagnosed by an expert radiologist on the basis of size, number, and shape of the lesions and presence of calcification of the lesions. 

### 2.2. Statistical Analysis

Continuous variables included age at diagnosis; body mass index (BMI); clinical tumor size (defined as the maximal tumor diameter on preoperative abdominal CT); and preoperative PLT count (10^9^/L) and serum Hb (g/dL), albumin (g/L), globulin (g/L), AST (U/L), and ALT (U/L) levels. Categorical variables included sex (male vs. female), Charlson comorbidity index (CCI) [14], presence of systemic symptoms (absent vs. present) (Table S1), clinical stage (defined according to the American Joint Committee on Cancer manual [15] and classified as cT1a vs. cT1b vs. cT2 vs. cT3–cT4), and clinical Nstage (defined according to the American Joint Committee on Cancer manual [15] and classified as cN0 vs. cN1).

A receiver operating characteristic (ROC) curve was generated to ascertain the cutoff value for clinical tumor size and albumin/globulin ratio (AGR). The clinical characteristics of the patients were compared using Student’s t-test (continuous variables) and the chi-square test or Fisher’s exact test (categorical variables). Logistic regression analyses were conducted to predict positive chest CT scans in patients with RCC selected for surgical treatment of kidney cancer to generate an odds ratio (OR) with a 95% confidence interval (CI). 

An 890-bootstrap resampling validation test [16] was performed to evaluate the concordance index (95% CI). Leave-one-out cross-validation [17] was performed to revise the concordance index for overfitting. To calculate the clinical power of the potential model, decision curve analysis was performed [18]. Then, to assess the number of potentially avoidable negative chest CT scans and number of eventually misdiagnosed positive chest CT scans, the clinical decision-making results were established based on a specific threshold-derived model. 

Statistical analyses were performed using Statistical Package for the Social Sciences version 16.0 for Windows (IBM Corp., Armonk, NY, USA) and RStudio 2022.07.1.554 for R software environment v.4.2.1 with the following libraries, packages, and scripts: moon-Book, Hmisc, plyr, stats, rms, graphics and dca. *p* < 0.05 was considered statistically significant.

### 2.3. Ethical Approval

The Institutional Review Board (IRB) of Kyungpook National University School of Medicine, Daegu, Republic of Korea (IRB no. KNUH 2022-03-009) approved this retrospective study. This trial was conducted in accordance with relevant laws and regulations, good clinical practices, and ethical principles, as described in the Declaration of Helsinki. The requirement for obtaining informed consent from all patients involved in this study was waived by our IRB owing to the retrospective nature of this trial. 

## 3. Results

Table 1 shows the basic clinical characteristics of the patients (n = 890) in the negative chest CT scan (n = 863) and positive chest CT scan (n = 27) groups. The overall rate of positive chest CT scans was 3.03% (n = 27). Only one patient had lung metastasis before surgery for cT1a, and 91 patients developed lung metastasis after surgery. The mean age at diagnosis was 60.29 ± 11.91 years. Men accounted for 67.1% (n = 597) of the patients. The mean BMI was 24.58 ± 3.61 kg/m^2^. The positive chest CT scan group showed significantly lower BMI (24.63 ± 3.59 vs. 23.05 ± 3.97, *p* = 0.025) than the negative chest CT scan group. The proportion of high CCI scores was significantly higher in the positive chest CT scan group than in the negative chest CT scan group (*p* = 0.022). In total, 503 (56.5%) patients had systemic symptoms (Table S1 [19]). The mean clinical tumor size was 47.77 ± 29.73 mm. The positive chest CT scan group had a significantly greater tumor size than the negative chest CT scan group (46.59 ± 28.40 vs. 85.70 ± 43.85, *p* < 0.001). The disease stage in 42 (4.7%) patients was cN1. The proportion of high cT and cN stages was significantly higher in the positive chest CT scan group than in the negative chest CT scan group (*p* < 0.001 and *p* = 0.001, respectively).

The mean preoperative PLT count (10^9^/L) (257.01 ± 78.31 vs. 335.74 ± 143.09, *p* = 0.008) and Hb level (g/dL) (13.66 ± 1.90 vs. 12.40 ± 2.85, *p* = 0.031) values were significantly different between the groups. The mean PLT/Hb ratio was 19.96 ± 10.75, and there were significant differences between the groups (19.65 ± 10.29 vs. 29.79 ± 18.35, *p* = 0.008). The mean preoperative serum albumin (g/L) (4.35 ± 0.39 vs. 3.99 ± 0.51, *p* = 0.001) and globulin (g/L) (3.02 ± 1.18 vs. 3.52 ± 0.55, *p* < 0.001) levels were significantly different between the groups. The mean AGR was 1.49 ± 0.30, and there were significant differences between the groups (1.50 ± 0.30 vs. 1.17 ± 0.24, *p* < 0.001). The mean preoperative serum AST and ALT levels and De Ritis ratio (AST/ALT) did not differ between the groups. The mean time to diagnosis of lung metastasis was 12.68 ± 17.50 months, and the mean follow-up period was 44.80 ± 30.19 months.

Table 2 shows the subgroup analyses of patients with cT1a and cT1b disease. In total, 18 and 28 patients with cT1a and cT1b disease, respectively, developed lung metastases after surgery (*p* = 0.001). The cN stage was significantly different between patients with cT1a and cT1b disease (*p* < 0.001). Further, there were significant differences in the mean AGR between patients with cT1a and cT1b disease (1.56 ± 0.27 vs. 1.50 ± 0.30, *p* = 0.003).

**Figure 1:**
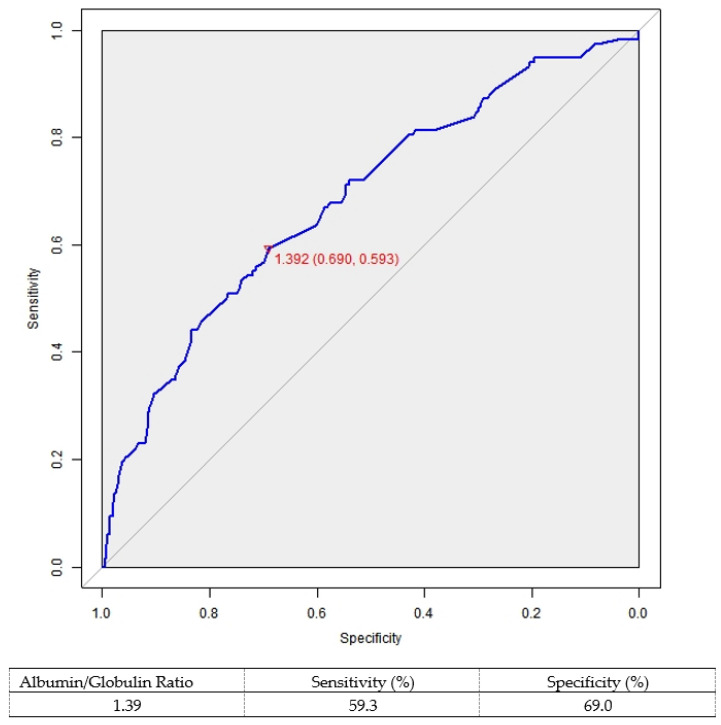
Receiver operator characteristic curve based on the albumin/globulin ratio.

The best cutoff value for AGR was 1.39 (sensitivity: 59.3%, specificity: 69.0%) in accordance with the ROC curve. The area under the ROC curve was 0.684 (95% CI: 0.631–0.737; *p* < 0.001; Figure 1).

The results of the multivariable logistic regression analysis are shown in Table 3. CCI was significantly associated with an increased risk of positive chest CT scan. CCI ≥4 (OR: 2.874; 95% CI: 1.437–5.757; *p* = 0.003) was associated with an increased risk of positive chest CT scans. cT1b (OR: 2.636; 95% CI: 1.412–4.921; *p* = 0.002), cT2 (OR: 4.103; 95% CI: 1.947–8.467; *p* < 0.001), and cT3–cT4 (OR: 13.847; 95% CI: 7.302–26.259; *p* < 0.001) were associated with an increased risk of positive chest CT scans when compared with cT1a. A low AGR (OR: 0.431; 95% CI: 0.197–0.941; *p* = 0.035) was also associated with an increased risk of positive chest CT scans.

**Figure 2:**
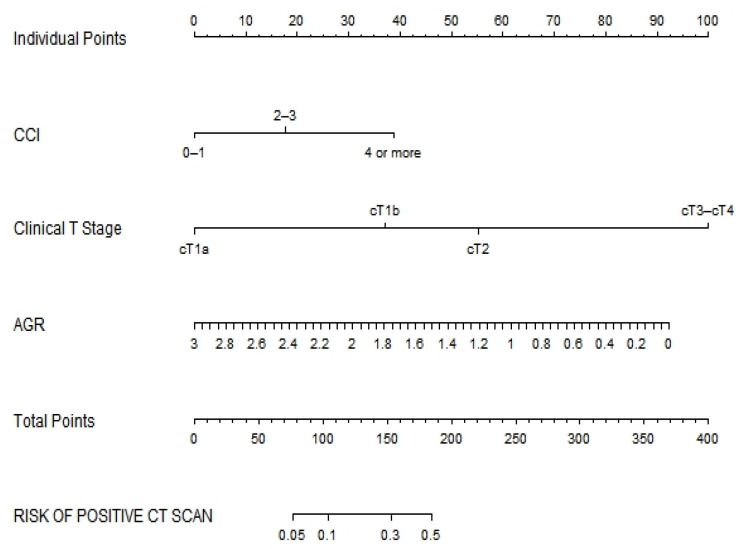
Nomogram predicting the risk of pulmonary metastasis in 890 patients with renal cell carcinoma who underwent nephrectomy at a single institution, 2011–2020. CCI, Charlson comorbidity index; AGR, albumin/globulin ratio.

After 890-sample bootstrap validation, the concordance index was 0.80 (95% CI: 0.758–0.850). The impact of each predictive factor based on the risk of a positive chest CT scan was plotted graphically by developing a nomogram (Figure 2).

**Figure 3:**
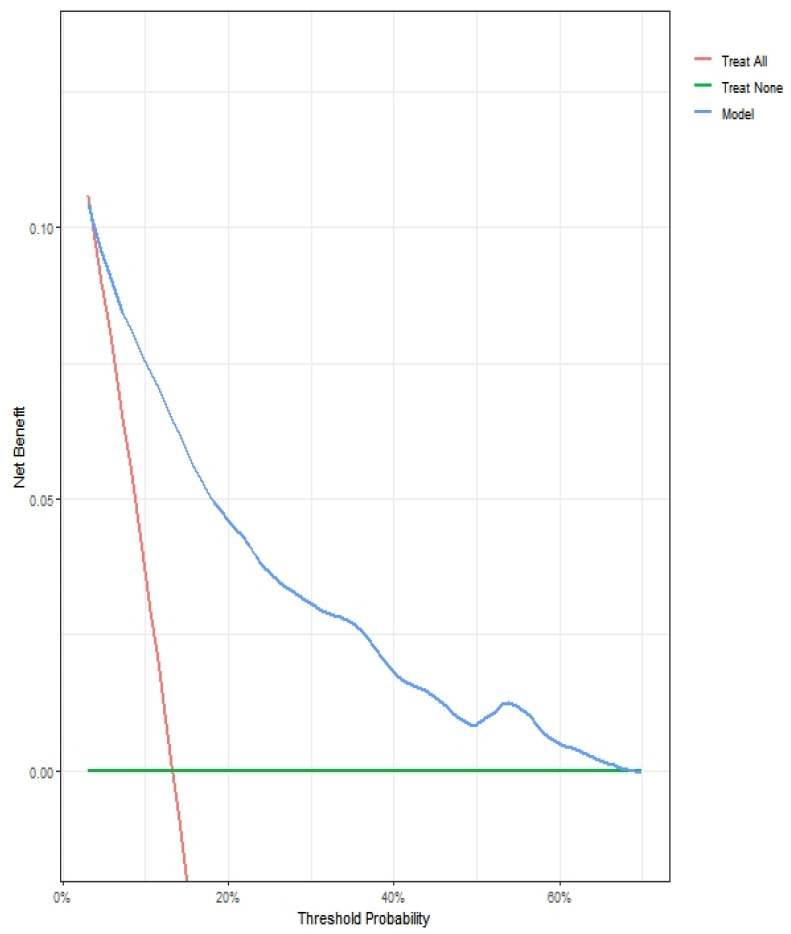
Two decision curve analyses delineating the clinical net benefit after using the potential model for the objective standard regarding when to perform preoperative chest computed tomography in 890 patients with renal cell carcinoma who underwent nephrectomy at a single institution, 2011–2020.

According to the decision curve analysis (Figure 3), the net benefit of the proposed strategy was superior to that of the select-all and select-none strategies. Therefore, when chest CT scans were performed with a risk of a positive result ≥10%, 532 (59.8%) negative chest CT scans could be prevented. Only 24 (2.7%) potentially positive chest CT findings were misdiagnosed.

## 4. Discussion

The hypothesis of the present study was that preoperative chest CT could be omitted in low-risk patients with RCC at initial diagnosis because of their relatively low risk of lung metastasis. We aimed to verify the risk of lung metastasis in patients with RCC scheduled to undergo surgical treatment to identify the objective indications for preoperative chest CT. Thus, we retrospectively analyzed data from a single tertiary institution to create a predictive model for assessing the risk of positive chest CT scans in the preoperative setting.

Ionizing radiation from X-rays can cause mutations in DNA. Most DNA damage is immediately restored; however, persistent DNA damage can cause cellular dysfunction, necrosis, and malignancy [20,21,22]. CT may have a higher radiation exposure risk than benefits [23]. The average radiation exposure dose from screening chest CT ranges from approximately 0.6 to 1.1 mSv per study [21]. According to the data from the Italung-CT trial, when additional follow-up chest CT is considered for indeterminate or suspicious pulmonary nodules, the 4-year cumulative effective dose can range from 3.3 to 5.8 mSv [23,24]. This radiation exposure is estimated to cause 11.7–20.5 radiation-induced lethal cancer cases per 100,000 50–70-year-old patients screened [21]. Accordingly, if chest CT screening for RCC cannot ensure a significant reduction in overall mortality due to lung metastasis, the harmful risks of radiation exposure associated with preoperative chest CT screening cannot be justified.

For patients with RCC, the initial and follow-up imaging modalities should be decided based on patient characteristics and recurrent patterns after the initial curative surgery as well as the recurrent risk evaluated at the time of initial treatment [11]. Regarding RCC, recurrence rates were 20–50%, even in patients with RCC who underwent partial or radical nephrectomy [25]. For metastatic RCC, the lung is the most frequent site of thoracic recurrence, and most cases of lung metastasis develop within 2–3 years of initial surgical treatment [7].

In patients with RCC, lung metastases primarily develop via hematogenous or lymphatic spread [26]. In 2019, Lee et al. investigated the patterns of thoracic recurrence from RCC following nephrectomy as a pilot study, including 39 patients who developed lung metastasis after nephrectomy [11]. They emphasized that the lower part of the lung had a greater distribution of parenchymal tissues and blood vessels than the upper part of the lung. Furthermore, lymphatic drainage usually passes through the thoracic duct from the retroperitoneal space to the mediastinal space. Thus, there is an increased possibility of detecting lung metastasis in the lower part of the lung because of the lung anatomy. Therefore, in most patients with intermediate- or high-risk RCC, initial recurrence or metastasis may develop in the abdomen or lower part of the lung. These aspects imply that only one abdominal CT covering thoracic spine level 7 is an effective imaging modality for patients with RCC with a history of nephrectomy.

In 2017, Larcher et al. verified that patients with ≥cT1b or cN1 RCC, presence of systemic symptoms, and high PLT/Hb ratio would benefit from preoperative staging chest CT to identify lung metastasis [12]. The rate of positive chest CT scans was 6% (n = 119), which was lower than that in the present study. Although this study was based on data over a wide time span from a single tertiary research institution (1987–2005) and patients had already been selected previously for local treatment, these patients cannot completely represent the general population. However, the large cohort size (n = 1946) makes this study meaningful. Despite the indicated differences between the present study and Larcher et al.’s study, the conclusions of both studies are similar. In 2020, Voss et al. [27] performed an external validation of the nomogram developed by Larcher et al. The authors proposed an easier and more simplified model and used more objective variables that are available for clinical application. The low-risk group from their model (tumor size ≤40 mm and no systemic symptoms) had a risk of positive chest CT scan of <1%, suggesting that preoperative chest CT can be omitted for these patients.

Numerous studies have shown that a high PLT/Hb ratio [28], low AGR [29,30,31], and high De Ritis ratio [32] may be associated with poor prognosis, not only for urologic malignancy but also for various tumors. If we narrow the scope only to the study of RCC, Peng et al. demonstrated that anemia and thrombocythemia were closely associated with clinicopathological features and were independent prognostic factors of cancer-specific survival in patients with RCC undergoing nephrectomy [33]. In 2017, Chen et al. [34] conducted a retrospective study that included 416 patients diagnosed with localized or locally advanced clear cell RCC. According to them, the best cutoff value for AGR was 1.22, lower than that used in the present study. The authors demonstrated that a low AGR was an independent predictive factor for estimating overall survival (hazard ratio [HR]: 6.53; 95% CI: 3.04–14.04; *p* < 0.001) and cancer-specific survival (HR: 8.81; 95% CI: 3.89–19.93; *p* < 0.001). Furthermore, Lee et al. [35] reported that an increased De Ritis ratio (AST/ALT) was significantly correlated with adverse postoperative outcomes in patients with localized clear cell RCC who underwent nephrectomy. Although the present study did not show any significant association between high PLT/Hb or De Ritis ratio and pulmonary metastasis, low AGR was significantly associated with a positive chest CT scan. These findings are consistent with those of a previous study published at our center [36].

In the present study, 118 (13.26%) patients had lung metastasis—91 (10.22%) patients developed lung metastases after surgery and 27 (3.03%) patients were diagnosed with lung metastases before surgery. Among the 27 patients with lung metastases diagnosed before surgery, only one (1/27, 3.7%) patient had cT1a disease. Therefore, we can assume the following medical situation. It can be assumed that patients with cT1a RCC and lung metastasis underwent nephrectomy without undergoing preoperative chest CT, and lung metastasis was discovered on chest CT after surgery. However, this situation does not appear to be worrisome. First, according to our study, this case was rare. Second, even if such a case occurs, there seems to be no problem in the treatment of patients with RCC since cytoreductive nephrectomy has already been performed. Although some studies, such as the Carmena study [37], have shown that cytoreductive nephrectomy before systemic therapy has insignificant effects on survival, the EAU [38] and NCCN [39] guidelines continue to recommend cytoreductive nephrectomy and metastasectomy.

Despite its novelty as the first study to focus on an Asian subpopulation and its clinically relative, large cohort, the present study has some limitations. These include retrospective data collection and single-center design. A retrospective design may introduce sampling bias. Furthermore, the definition of detection of lung metastasis, which was the primary endpoint, was solely based on clinical suspicion without histological confirmation, such as lung biopsy. Excluding patients with multiple metastases (not only in the lungs) is also a limitation of the present study. This may underestimate the value of preoperative CT in obtaining reliable clinical tumor–node–metastasis staging, which can lead to an incomplete observational study of the entire RCC spectrum in South Korea. Further large-scale, population-based, prospective, multi-institutional studies are necessary in the near future to confirm our study findings.

## 5. Conclusions

The present study demonstrated assessment of the risk of lung metastasis in Asian patients with RCC using preoperative patient characteristics and imaging modalities with optimum predictive accuracy. We conclude that performing chest CT is not necessary in all cases. We recommend that patients with cT stage ≥cT1b, CCI ≥4, or AGR <1.39 should be chosen for preoperative chest CT, and patients with cT1a disease can be excluded from preoperative chest CT screening for RCC staging. Based on these strategies, a negative chest CT scan can be prevented in 59.8% of cases, whereas a positive chest CT scan can be missed in only 2.7% of cases. Future research providing external validation with a large cohort will support the widespread use of these proposed models during clinical decision- making in patients with RCC before radical surgery.

## Figures and Tables

**Table 1:** Clinical characteristics of patients.

Variable	Overall	Negative Chest CT Scan Prior to Surgery	Positive Chest CT Scan Prior to Surgery	*p* Value
Population, n (%)	890 (100.00)	863 (96.97)	27 (3.03)	
Positive chest CT scan after surgery				<0.001
No	772 (86.74)	772 (89.46)	0 (0.0)	
Yes	118 (13.26)	91 (10.54)	27 (100.00)	
Age, years	60.29 ± 11.91	60.28 ± 11.91	60.81 ± 12.12	0.818
Sex, n (%)				0.320
Male	597 (67.1)	576 (66.74)	21 (77.78)	
Female	293 (32.9)	287 (33.26)	6 (22.22)	
BMI, kg/m^2^	24.58 ± 3.61	24.63 ± 3.59	23.05 ± 3.97	0.025
CCI, n (%)				0.022
0–1	185 (20.79)	183 (21.21)	2 (7.41)	
2–3	363 (40.79)	355 (41.14)	8 (29.63)	
≥4	342 (38.43)	325 (37.66)	17 (62.96)	
Systemic symptoms, n (%)				0.999
Absent	387 (43.5)	375 (43.45)	12 (44.44)	
Present	503 (56.5)	488 (56.55)	15 (55.56)	
Clinical size, mm	47.77 ± 29.73	46.59 ± 28.40	85.70 ± 43.85	<0.001
Clinical T stage, n (%)				<0.001
cT1a	424 (47.6)	423 (49.02)	1 (3.70)	
cT1b	251 (28.2)	247 (28.62)	4 (14.81)	
cT2	98 (11.0)	95 (11.01)	3 (11.11)	
cT3–cT4	117 (13.1)	98 (11.36)	19 (70.37)	
Clinical N stage, n (%)				0.001
cN0	848 (95.3)	827 (95.83)	21 (77.78)	
cN1	42 (4.7)	36 (4.17)	6 (22.22)	
Preoperative PLT, 10^9^/L	259.40 ± 82.02	257.01 ± 78.31	335.74 ± 143.09	0.008
Preoperative Hb, g/dL	13.62 ± 1.95	13.66 ± 1.90	12.40 ± 2.85	0.031
PLT/Hb ratio	19.96 ± 10.75	19.65 ± 10.29	29.79 ± 18.35	0.008
Serum albumin (g/L)	4.34 ± 0.40	4.35 ± 0.39	3.99 ± 0.51	0.001
Serum globulin (g/L)	3.03 ± 1.17	3.02 ± 1.18	3.52 ± 0.55	<0.001
AGR	1.49 ± 0.30	1.50 ± 0.30	1.17 ± 0.24	<0.001
AST	26.93 ± 19.17	26.98 ± 19.34	25.41 ± 12.36	0.528
ALT	24.64 ± 17.25	24.69 ± 17.33	23.00 ± 14.75	0.616
De Ritis Ratio	1.27 ± 0.67	1.27 ± 0.67	1.25 ± 0.46	0.801
Time to diagnosis of lung metastasis (months)	12.68 ± 17.50	16.49 ± 18.32 (only 91 patients)	0.00 ± 0.00	<0.001
Follow-up period (months)	44.80 ± 30.19	45.56 ± 30.00	20.48 ± 26.44	<0.001

BMI, body mass index; CCI, Charlson comorbidity index; PLT, platelet; Hb, hemoglobin; AGR, albumin/globulin ratio; AST, aspartate transaminase; ALT, alanine aminotransferase; CT, computed tomography.

**Table 2:** Subgroup analysis of only patients with clinical T stages cT1a and cT1b.

Variable	Overall	cT1a	cT1b	*p* Value
Population, n (%)	675 (100.00)	424 (62.81)	251 (37.19)	
Positive chest CT scan prior to surgery				0.066
No	670 (99.26)	423 (99.76)	247 (98.41)	
Yes	5 (0.74)	1 (0.24)	4 (1.59)	
Positive chest CT scan after surgery				0.001
No	629 (93.19%)	406 (95.75)	223 (88.84)	
Yes	46 (6.81%)	18 (4.25)	28 (11.16)	
Age, years	60.39 ± 11.62	60.74 ± 12.00	59.81 ± 10.96	0.318
Sex, n (%)				0.283
Male	454 (67.26%)	292 (68.87)	162 (64.54)	
Female	221 (32.74%)	132 (31.13)	89 (35.46)	
BMI, kg/m^2^	24.68 ± 3.64	24.54 ± 3.66	24.91 ± 3.61	0.207
CCI, n (%)				0.514
0–1	142 (21.04)	92 (21.70)	50 (19.92)	
2–3	282 (41.78)	170 (40.09)	112 (44.62)	
≥ 4	251 (37.19)	162 (38.21)	89 (35.46)	
Systemic symptoms, n (%)				0.883
Absent	292 (43.26%)	182 (42.92)	110 (43.82)	
Present	383 (56.74%)	242 (57.08)	141 (56.18)	
Clinical size, mm	35.90 ± 15.96	26.14 ± 10.03	52.37 ± 9.07	<0.001
Clinical N stage, n (%)				<0.001
cN0	661 (97.93%)	422 (99.53)	239 (95.22)	
cN1	14 (2.07%)	2 (0.47)	12 (4.78)	
Preoperative PLT, 10^9^/L	250.54 ± 72.62	243.22 ± 70.70	262.92 ± 74.25	0.001
Preoperative Hb, g/dL	13.80 ± 1.79	13.88 ± 1.77	13.66 ± 1.82	0.131
PLT/Hb ratio	18.89 ± 10.03	18.27 ± 10.90	19.92 ± 8.28	0.027
Serum albumin (g/L)	4.39 ± 0.36	4.42 ± 0.34	4.35 ± 0.39	0.014
Serum globulin (g/L)	2.91 ± 0.48	2.88 ± 0.45	2.98 ± 0.52	0.011
AGR	1.54 ± 0.28	1.56 ± 0.27	1.50 ± 0.30	0.003
AST	27.80 ± 20.97	27.73 ± 20.27	27.92 ± 22.16	0.910
ALT	25.65 ± 17.98	26.14 ± 18.12	24.82 ± 17.74	0.354
De Ritis Ratio	1.25 ± 0.70	1.25 ± 0.77	1.26 ± 0.58	0.835
Time to diagnosis of lung metastasis (months)	17.20 ± 20.18	15.06 ± 16.94	18.50 ± 22.11	0.585
Follow-up period (months)	45.13 ± 30.04	43.43 ± 27.98	48.00 ± 33.08	0.068

BMI, body mass index; CCI, Charlson comorbidity index; PLT, platelet; Hb, hemoglobin; AGR, albumin/globulin ratio; AST, aspartate transaminase; ALT, alanine aminotransferase; CT, computed tomography.

**Table 3:** Multivariable logistic regression analysis predicting positive chest computed tomography scans in patients with renal cell carcinoma selected for surgical treatment for kidney cancer.

Predictor	Multivariable Analysis	
	OR (95% CI)	*p* Value
BMI	0.970 (0.893–1.054)	0.472
CCI, n (%)		
0–1	1.000 (reference)	
2–3	1.618 (0.791–3.308)	0.188
≥4	2.874 (1.437–5.747)	0.003
Clinical T stage, n (%)		
cT1a	1.000 (reference)	
cT1b	2.636 (1.412–4.921)	0.002
cT2	4.103 (1.947–8.647)	<0.001
cT3–cT4	13.847 (7.302–26.259)	<0.001
Clinical N stage, n (%)		
cN0	1.000 (reference)	
cN1	1.868 (0.871–4.004)	0.108
PLT/Hb ratio	1.013 (0.993–1.034)	0.207
AGR	0.431 (0.197–0.941)	0.035

OR, odds ratio; BMI, body mass index; CCI, Charlson comorbidity index; PLT, platelet; Hb, hemoglobin; AGR, albumin/globulin ratio.

## Data Availability

All relevant data are included in the manuscript and its Supporting Information Files.

## Supplementary Materials

The following supporting information can be downloaded from: https://www.mdpi.com/article/10.3390/cancers14225558/s1, Table S1: Definition of systemic symptoms.
