# Supplementary material for: Should We Always Perform Preoperative Chest Computed Tomography in Patients with cT1a Renal Cell Carcinoma?
Source: Cancers (Basel). 2022 Nov 12;14(22):5558. doi: 10.3390/cancers14225558 (PMC9688927; doi:10.3390/cancers14225558)
Supplement: Supplementary file 1 [file cancers-14-05558-s001.zip › cancers-1915751-supplementary.pdf]

Table S1. Definition of systemic symptoms.

**Systemic symptoms**
Fever or chills
Weight loss
Sweat
Hypertension
Cachexia
Dyspnea
Pain
Anorexia
